# Delayed Recognition of Disorders of Sex Development (DSD): A Missed Opportunity for Early Diagnosis of Malignant Germ Cell Tumors

**DOI:** 10.1155/2012/671209

**Published:** 2012-01-19

**Authors:** Remko Hersmus, Hans Stoop, Stefan J. White, Stenvert L. S. Drop, J. Wolter Oosterhuis, Luca Incrocci, Katja P. Wolffenbuttel, Leendert H. J. Looijenga

**Affiliations:** ^1^Department of Pathology, Erasmus MC-University Medical Center Rotterdam, Josephine Nefkens Institute, Daniel den Hoed Cancer Center, P.O. Box 2040, 3000 CA Rotterdam, The Netherlands; ^2^Centre for Reproduction and Development, Monash Institute of Medical Research, Melbourne, VIC, Australia; ^3^Department of Pediatric Endocrinology, Erasmus MC-University Medical Center Rotterdam, Sophia, Rotterdam, The Netherlands; ^4^Department of Radiation Oncology, Erasmus MC-University Medical Center Rotterdam, Daniel den Hoed Cancer Center, Rotterdam, The Netherlands; ^5^Department of Pediatric Urology, Erasmus MC-University Medical Center Rotterdam, Sophia, Rotterdam, The Netherlands

## Abstract

Disorders of sex development (DSD) are defined as a congenital condition in which development of chromosomal, gonadal or anatomical sex is atypical. DSD patients with gonadal dysgenesis or hypovirilization, containing part of the Y chromosome (GBY), have an increased risk for malignant type II germ cell tumors (GCTs: seminomas and nonseminomas). DSD may be diagnosed in newborns (e.g., ambiguous genitalia), or later in life, even at or after puberty. Here we describe three independent male patients with a GCT; two were retrospectively recognized as DSD, based on the histological identification of both carcinoma *in situ* and gonadoblastoma in a single gonad as the cancer precursor. Hypospadias and cryptorchidism in their history are consistent with this conclusion. The power of recognition of these parameters is demonstrated by the third patient, in which the precursor lesion was diagnosed before progression to invasiveness. Early recognition based on these clinical parameters could have prevented development of (metastatic) cancer, to be treated by systemic therapy. All three patients showed a normal male 46,XY karyotype, without obvious genetic rearrangements by high-resolution whole-genome copy number analysis. These cases demonstrate overlap between DSD and the so-called testicular dysgenesis syndrome (TDS), of significant relevance for identification of individuals at increased risk for development of a malignant GCT.

## 1. Introduction

Congenital conditions in which development of chromosomal, gonadal, or anatomical sex is atypical are termed “Disorders of Sex Development” (DSD) [1] and have replaced the formerly used “intersex” term. It is estimated that DSD affects 1 in 4,500 to 5,000 live births in the general population, although with variability regarding the various DSD subtypes [1]. DSD patients are subdivided into different entities: 46,XY DSD, 46,XX DSD, and sex chromosomal DSD. Within these subgroups, patients, with gonadal dysgenesis (GD) and hypovirilization with presence of part of the Y chromosome (i.e., GBY), are known to have an increased risk to develop carcinoma *in situ* (CIS) or gonadoblastoma (GB), the precursor lesions of seminoma(SE)/dysgerminoma(DG) and nonseminoma, referred to as malignant type II germ cell tumors (GCTs) ([2–4], for review). In GD migration of the germ cells and/or their organization in the gonads is disturbed, leading to incomplete formation of the gonads. Hypovirilization is caused by defects in androgen-dependent target tissues, errors in testosterone biosynthesis, and testicular unresponsiveness to stimulation from the pituitary [5], leading to underdevelopment of the male differentiation lineage.

GB is the *in situ* germ cell malignancy of the ovary and dysgenetic gonad which, in a significant number of cases, will develop into an invasive dysgerminoma or, less often, nondysgerminoma, being histologically and genetically counterparts of testicular seminoma and nonseminoma [6]. GB is composed of a mixture of embryonic germ cells (OCT3/4 and SCF (official term: KITLG) positive, amongst others) and supportive cells, with characteristics of granulosa cells (FOXL2 positive) [7]. GB can be found in undifferentiated gonadal tissue and in gonadal tissue with immature testis differentiation [8], overall related to a low level of testicularization (i.e., level of testis formation). CIS (cells also positive for OCT3/4 and SCF, amongst others), on the other hand, being the precursor of the similar types of cancer (SE and nonseminoma) of the testis, is associated with SOX9-positive Sertoli cells [7] and is found in well-differentiated testicular tissue [9].

For malignant transformation of embryonic germ cells in the context of type II GCTs, presence of part of the Y chromosome is crucial, referred to as gonadoblastoma on the Y chromosome (GBY) region by Page in 1987 [2]. *TSPY* is currently considered to be the most likely candidate gene for this genomic region [10, 11], and of diagnostic value, because both CIS and GB show coexpression of OCT3/4, SCF, and TSPY.

In spite of the overall low incidence in the general population, type II testicular GCTs are the most common malignancy in Caucasian males aged between 15 and 45 years, the incidence of which is still rising [12]. It has been suggested that the so-called testicular dysgenesis syndrome (TDS) is the underlying reason [13], estimated to affect 1 in 500 live births. However, existence of TDS is also questioned [14]. TDS links various clinical observations like cryptorchidism, subfertility/infertility, and hypospadias with exposure to certain environmental factors, with either a xenoestrogen or antiandrogen function. However, genetic factors, especially a limited number of single nucleotide polymorphisms (SNPs) are also recognized to play a role in development of this type of cancer [15, 16]. Most likely, the pathogenesis is a close and subtle interplay between both genetic and environmental factors, referred by us to as “Genvironment.”

Here three unique unrelated male patients are presented demonstrating the relevance of TDS and DSD recognition for early diagnosis of malignant type II GCTs, possibly preventing progression to metastasized disease.

## 2. Materials and Methods 

### 2.1. Patients

Three unrelated male patients, all with hypospadias and cryptorchidism in their clinical history, are described. All patients underwent hypospadias corrections, and two patients had orchidopexy early in life. Two of the patients were only retrospectively recognized as having DSD based on the presence in a single gonad of GB next to CIS as precursor lesions. The third patient described, having been recognized early in life as having DSD/TDS (i.e., hypospadias and cryptorchidism), shows that early identification of the condition can lead to early detection of the cancer precursor lesion before progression to invasiveness occurs. Detailed description is presented in Section 3.

### 2.2. Tissue Samples

Collected tissue samples were diagnosed according to WHO standards [17] by an experienced pathologist in gonadal pathology, including GCTs (JWO). The use of tissue samples for scientific reasons was approved by an institutional review board (MEC 02.981 and CCR2041). Samples were used according to the “Code for Proper Secondary Use of Human Tissue in The Netherlands” as developed by the Dutch Federation of Medical Scientific Societies (FMWV, Version 2002, updated 2011). Fresh tissue material was fixed in 10% buffered formalin for 24 hrs and paraffin embedded according to standard protocols.

### 2.3. Immunohistochemical Staining

Immunohistochemistry was performed on paraffin-embedded tissue sections of 3-*μ*m thickness. Hematoxylin (Klinipath, Duiven, The Netherlands) and eosin (Klinipath) counterstaining was performed according to standard procedures. After deparaffinization and 5 min incubation in 3%  H_2_O_2_ to inactivate endogenous peroxidase activity, antigen retrieval was carried out by heating under pressure of up to 1.2 bar in an appropriate buffer; 0.01 M sodium citrate (pH 6) or 0.01 M EGTA, 0.01 M TRIS (pH 9). After blocking endogenous biotin using the avidin/biotin blocking kit (SP-2001, Vector Laboratories, Burlingame, CA, USA), the sections were incubated for either 2 hrs at room temperature (OCT3/4, SOX9) or overnight at 4°C (TSPY, FOXL2, SCF). Appropriate biotinylated secondary antibodies were used for detection and were visualized using the avidin-biotin detection and substrate kits (Vector Laboratories). The antibodies used directed against OCT3/4, TSPY, SCF, SOX9, and FOXL2 have been described before [7, 18–20].

### 2.4. Fluorescent In Situ Hybridization

 Slides of 5 *μ*m thickness were deparaffinized and heated under pressure of up to 1.2 bar in appropriate buffer; 0.01 M sodium citrate (pH 6). Slides were digested using 0.01% pepsin (Sigma Aldrich, St. Louis, MO, USA) in 0.02 M HCl at 37°C, with an optimal digestion time of 2.5 min. Slides were rinsed and dehydrated, and the probes dissolved in hybridization mixture were applied. Probes for centromere X (BamHI) and centromere Y (DYZ3) were used, labeled with digoxigenin-11-dUTP and biotin-16-dUTP (Roche Diagnostics, Mannheim, Germany) using a nick-translation kit (Gibco BRL, Paisley, UK). After denaturizing (80°C for 10 min), hybridization overnight (37°C), and washing steps, probes were visualized using Cy3-conjugated avidin (1 : 100, Jackson ImmunoResearch, West Grove, PA, USA) and Sheep-anti-dig FITC (1 : 50, Roche Diagnostics) and analyzed using a fluorescent microscope (Leica Microsystems, Rijswijk, The Netherlands).

### 2.5. Copy Number Analysis

Genomic DNA was isolated from peripheral blood (patient 1 and 3) and frozen gonadal tissue without presence of malignant cells (patient 2) using standard procedures. For each sample, 200 ng of DNA was labelled and hybridized onto the Human OmniExpress microarray (Illumina, San Diego, CA, USA) at the Australian Genome Research Facility (Melbourne, Australia) following manufacturer's instructions. Data was analyzed with Genome Studio (Illumina) and cnvPartition, using default settings.

## 3. Results

### 3.1. Clinical History, Hormonal and Genetic Data, and Immunohistochemical Analyses

**Patient 1**

Review of the existing clinical data was prompted by the histological evaluation of the right testis at the age of 26 years (showing dysgenetic characteristics, see below). It was found that the patient had multiple surgical corrections of proximal hypospadias between his second and tenth year of age, because of the severity of this anomaly. Orchidopexy of the left testis by an inguinal approach was performed at three years of age, while no right gonad was found during inguinal exploration on the right side at that time. At 26 years of age, the patient underwent surgery for a left-sided inguinal hernia. During the procedure, the right testis (inguinal position) was identified at the left hand side (i.e., crossed testicular ectopia) and removed because of a macroscopically abnormal/tumor-like appearance.

**Figure 1:**
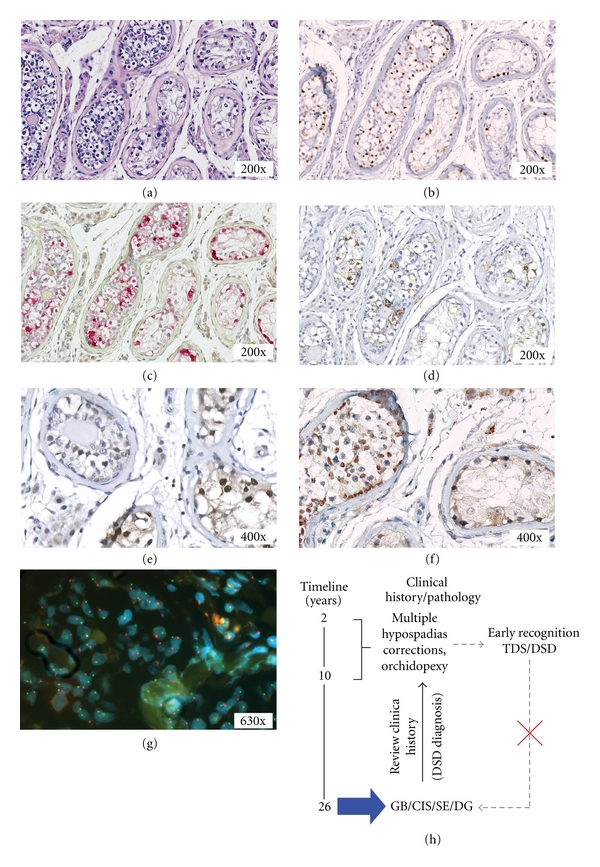
Immunohistochemical staining and fluorescent *in situ* hybridization (FISH) of the gonadoblastoma and carcinoma *in situ* lesions of patient 1. (a) Representative hematoxylin and eosin staining. The germ cells present in the GB and CIS stain positive for (b) OCT3/4 (brown), (c) TSPY (red), and (d) SCF (brown). (e) The supportive cells in the CIS lesion are SOX9 positive (brown staining) and are negative for FOXL2. (f) In the GB, the supportive cells stain positive for FOXL2 (brown staining) and are negative for SOX9. (a–f) In every image the GB lesion is shown on the left side (embryonic germ cells intermixed with granulose-like supportive cells), CIS containing seminiferous tubules on the right side (CIS cells associated with Sertoli cells on the basal lamina). Magnification 200x and 400x for all. Slides (b)–(f) are counterstained with hematoxylin. (g) Representative FISH with Y-centromere-specific probe (shown in red) and X-centomere-specific probe (shown in green). Magnification 630x. (h) Schematic representation of the different moments in time of clinical intervention, blue arrow, identification of a malignant type II germ cell tumor, together with GB and CIS as precursor lesions at the age of 26 years. Review of the clinical history showed hypospadias and cryptorchid testes, signs of TDS/DSD which were not recognized at an early age. Grey-dashed arrows; early recognition of TDS/DSD could have allowed early detection and treatment of the malignancy, thereby, preventing the need for additional systemic treatment.

Histological examination of this gonad showed dysgenetic characteristics, containing CIS, GB, DG, and SE (representative hematoxylin and eosin (H&E) staining shown in Figure 1(a)). The CIS- and GB-germ cells showed a positive staining for OCT3/4 (Figure 1(b), brown), TSPY (Figure 1(c), red), and SCF (Figure 1(d), brown). The supportive cells in context of CIS stained predominantly positive for SOX9 (Figure 1(e), brown), while those in the context of GB stained predominantly for FOXL2 (Figure 1(f), brown). Coexpression is, however, observed, suggesting an issue of balance. In line with current treatment options, the patient received prophylactic radiotherapy according to standard guidelines. During close followup (3 years), the patient showed no relapse of the disease.

Genetic analysis by karyotyping of peripheral blood lymphocytes, and FISH using X and Y centromeric probes on gonadal tissue (representative FISH shown in Figure 1(g)) indicated a normal male 46,XY constitution. Hormonal data analysis at the age of 24 years indicated a suboptimal testicular function (hypergonadotropic hypogonadism), FSH 12 and 17.5 U/L (normal 2.0–7.0 U/L), LH 5.6 and 8.4 U/L (normal 1.5–8.0 U/L), testosterone 13.2 and 16.2 (normal 10–30 nmol/L), Inhibin B 119 and 74 ng/L (normal 150–400 ng/L). Tumor markers measured after the removal of the affected gonad with the cancer showed a slightly elevated level of AFP 15–19 *μ*g/L (normal < 10–15 *μ*g/L), normal levels of *β*-HCG < 0.5 IU/L (normal < 0.5 IU/L), and LDH 152–314 U/L (normal < 450 U/L).

Taken together, the histological observations, clinical history, karyotyping, and hormonal data support the diagnosis of the patient as a 46,XY DSD, type A: disorder of testicular development, 1: partial gonadal dysgenesis [1]. A summary of the various actions and observations is schematically shown in Figure 1(h).

**Patient 2**

Review of the (limited) clinical history was provoked by the histological evaluation of the left testis at the age of 21 years, showing dysgenetic characteristics (see below). It revealed presence of bilateral intra-abdominal testes, while the male patient also showed hypospadias, as well as presence of a uterus. At 20 years of age, the patient was diagnosed with a right intra-abdominal testicular SE (of which no material or further information could be retrieved). During surgical removal of the affected gonad, the left sided intra-abdominal testis was positioned at an inguinal site. This remaining testis was biopsied six months later because of unexplained enlargement and was subsequently removed because of presence of CIS and GB (see below).

**Figure 2:**
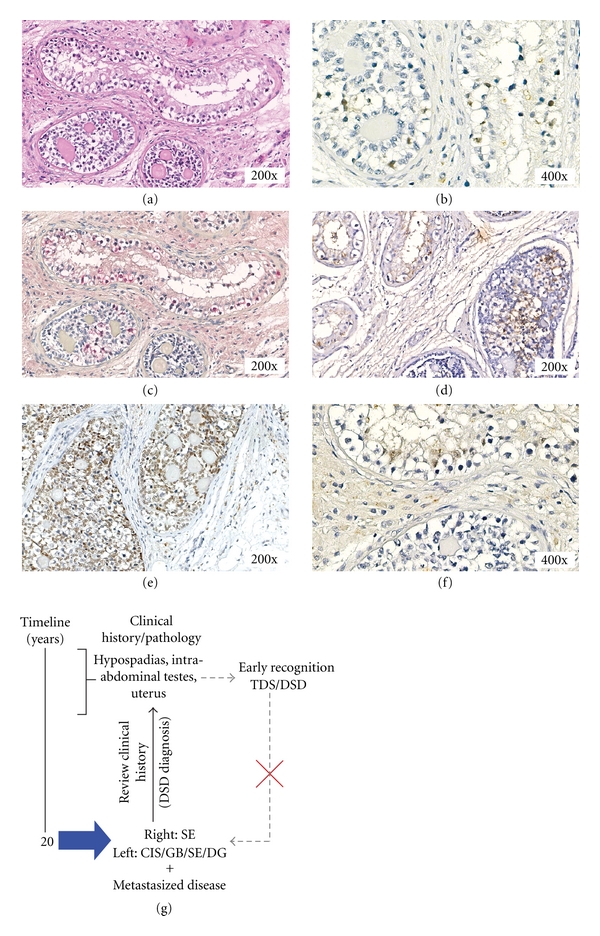
Immunohistochemical staining of the gonadoblastoma and carcinoma *in situ* lesions of patient 2. (a) Representative hematoxylin and eosin staining. Positive staining for (b) OCT3/4 (brown), (c) TSPY (red), and (d) SCF (brown) of the germ cells present in the GB and CIS. (e) In the GB the supportive cells stain positive for FOXL2 (brown). (f) The supportive cells in the CIS lesion are SOX9 positive (brown staining) and are negative for FOXL2. (a–d), (f) Again, both GB (embryonic germ cells intermixed with granulose-like supportive cells) and CIS (associated with Sertoli cells on the basal membrane of the tubules) are shown. Magnification 200x and 400x for all. Slides (b)–(f) are counterstained with hematoxylin. (g) Timeline showing the clinical history, histology, and actions taken.

Histological evaluation of the left biopsy showed the presence of GB and CIS, which was followed by orchidectomy. Further histological examination indicated the presence of dysgenetic characteristics, CIS and GB (representative H&E shown in Figure 2(a)), supported by staining for OCT3/4 (Figure 2(b), brown), TSPY (Figure 2(c), red), and SCF (Figure 2(d), brown), next to SE and DG. The supportive cells in GB stained again positive for FOXL2 (Figure 2(e), brown) and for SOX9 in CIS (Figure 2(f), brown). Because of proven metastasized disease, the patient received chemotherapy following standard procedures. No follow-up information is available.

Genetic analysis by karyotyping of peripheral blood lymphocytes and FISH using X and Y centromeric probes on gonadal tissue indicated a normal male 46,XY karyotype (data not shown). No hormonal or tumor marker data was available.

In summary, histological evaluation, review of clinical history, and karyotyping indicate that the patient must be diagnosed as a 46,XY DSD, type A: disorder of testicular development, 1: partial gonadal dysgenesis [1]. A summary of the various actions and observations is schematically shown in Figure 2(g).

**Patient 3**

**Figure 3:**
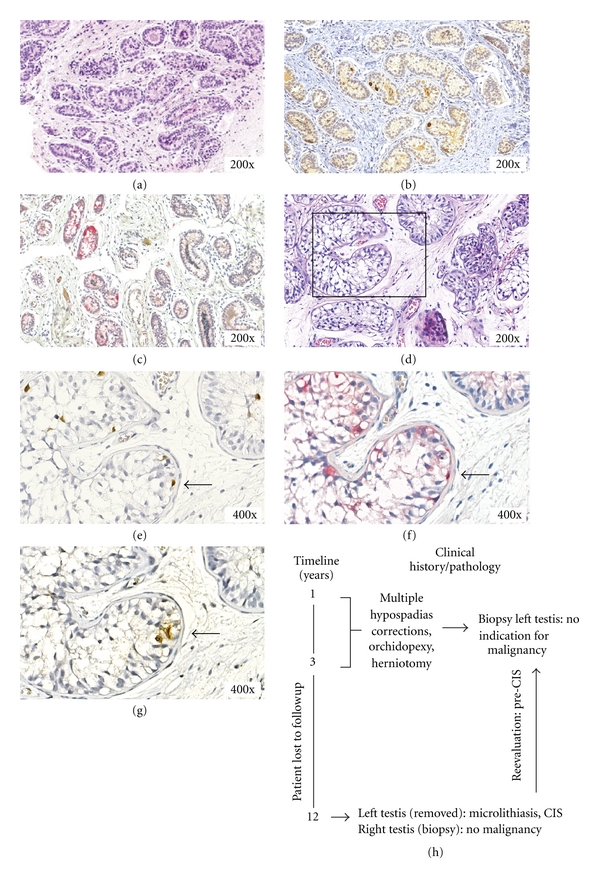
Immunohistochemical staining of the carcinoma *in situ* lesion of patient 3 at three and twelve years of age. (a) Representative hematoxylin and eosin staining. Positive staining for (b) OCT3/4 (brown), (c) TSPY (red) of the germ cells present in the CIS. (a–c) Biopsy tissue at 3 years of age. (d) Representative hematoxylin and eosin staining. Positive staining for (e) OCT3/4 (brown), (f) TSPY (red), and (g) SCF (brown) of the CIS cells. (d–g) Gonadal tissue at 12 years of age. (e–g) Region indicated with a square in (d) is shown. Note the expression of OCT3/4, TSPY, and SCF in the CIS cell indicated by the arrow. Magnification 200x and 400x for all. Slides (b)–(g) are counterstained with hematoxylin. (h) Timeline showing the clinical history, histology, and actions taken.

The male patient showed multiple congenital anomalies at birth, amongst other penoscrotal hypospadias and bilaterally cryptorchid testes. He underwent multiple hypospadias corrections at one and two years of age. Orchidopexy of the right testis to a high scrotal position was performed at two years of age, and a herniotomy and orchidopexy, to a high scrotal position, of the left testis was carried out at 3 years of age. Overall appearance of the left testis together with total dissociation of epididymis and testis prompted a biopsy to be taken at that time (representative H&E shown in Figure 3(a)). It was diagnosed as prepubertal testicular parenchyma with seminiferous tubules containing Sertoli cells and germ cells, without indication for malignancy. The patient was lost to followup until 12 years of age at which time he was examined because of incontinence problems and came under attention of the initial clinician treating the hypospadias by coincidence. Physical examination showed a pubertal boy (Tanner stage P4G3) with a scrotal localization of the right testis, while the left testis was not palpable. Further examination using ultrasound showed an inguinal position of the left testis (ascending testis) and bilateral testicular microcalcifications (microlithiasis). Because of the inability to position the left testis in the scrotum, and the knowledge about the increased risk for development of a malignant GCT based on the clinical characteristics, the left testicle was removed, and the right testis was biopsied.

Histological examination of the left testis (representative H&E staining shown in Figure 3(d)) showed seminiferous tubules containing CIS, supported by staining for OCT3/4 (Figure 3(e), brown), TSPY (Figure 3(f), red), and SCF (Figure 3(g), brown). Costaining of these markers in single CIS cells was identified (indicated by the arrow). The presence of CIS initiated reexamination of the biopsy taken at the age of three years. Because of limited material available, only staining for OCT3/4 (Figure 3(b), brown) and TSPY (Figure 3(c), red) could be done, showing the presence of premalignant germ cells, referred to as pre-CIS. This conclusion was not made at the time of original sampling because of lack of appropriate markers. The biopsy taken from the right testis showed normal testicular parenchyma without malignancy (negative OCT3/4 staining, data not shown).

Genetic analysis by karyotyping of peripheral blood lymphocytes and FISH using X and Y centromeric probes on gonadal tissue indicated a normal male 46,XY karyotype (data not shown). Hormonal data at the age of 12 years were as follows: FSH 1.8 and 3.1 U/L (normal < 6.0 U/L), LH 0.4 and 1.0 U/L (normal < 2.5 U/L), testosterone 1.6 and 6.7 nmol/L (normal 3.0–6.5 nmol/L), and AMH 18.8 *μ*g/L (normal 30–200 *μ*g/L). The tumor markers tested were within the normal range: AFP < 1 *μ*g/L (normal < 10 *μ*g/L) and *β*-HCG 0.1 IU/L (normal < 0.5 IU/L).

Taken together, histological evaluation, review of clinical history, and karyotyping indicate that the patient must be diagnosed as 46,XY DSD, type A: disorder of testicular development, 1: partial gonadal dysgenesis [1]. A summary of the various actions and observations in time is schematically shown in Figure 3(h).

### 3.2. Copy Number Analysis of Known DSD Genes

A peripheral blood DNA sample from patient 1 and 3 and a DNA sample isolated from frozen gonadal tissue from patient 2 (as no peripheral blood was available) were checked for copy number changes by genome-wide SNP analysis using Illumina OmniExpress Beadchips. This supported the 46,XY karyotype and showed no aberrations affecting known DSD genes (data not shown).

## 4. Discussion

Here, two unrelated male patients are presented, both diagnosed with an invasive malignant type II GCT. One was prophylactically treated with irradiation for a stage I seminoma, and the other received chemotherapy for proven metastasized disease. These treatment protocols have been found to increase the risk for long-term sequelae [21]. Presence of GB, known to be associated with DSD [8], besides CIS, as precursor in these patients, triggered review of their clinical history. Both cases showed severe hypospadias and cryptorchidism. These are identifiers of DSD, as well as TDS, both conditions known for their increased risk of malignant type II GCTs [3, 13]. In addition, patient 1 had crossed testicular ectopia, a very rare anomaly, reported to be associated with TDS and DSD [22]. No genetic confirmation of an underlying DSD was found in any of the patients, even using high-resolution genome wide analysis. In spite of this lack of identification of the molecular basis of the underlying disorder, the observations have significant implications regarding development of strategies for early diagnosis of type II GCTs, as well as understanding the biology of the disease.

DSD patients can be diagnosed early in life based on various characteristics, including sexual ambiguity, family history, discordant karyotype and genital appearance, and aberrant male and female genitalia. In children and young adults, however, DSD can present as an inguinal hernia in a girl, incomplete or delayed puberty, virilization in a girl, primary amenorrhea, breast development in a boy, and a previously unrecognized genital ambiguity [1].

When sex determination is disrupted in an early stage of Sertoli cell differentiation, a high risk for GB is found [9]. The GB lesion is composed of immature germ cells intermixed with supportive cells classified as granulosa [23]. The GB lesions found in the two presented patients showed these characteristics as well, based on immunohistochemical finding using OCT3/4, TSPY, SCF, SOX9, and FOXL2. Next to the GB component, CIS was also present in both. This in fact triggered the search for additional clinical arguments in line with the diagnose of these patients as DSD. The findings presented indicate that, by proper application of the current knowledge of risk factors for type II GCTs, these patients could have been diagnosed earlier, thereby, possibly preventing the use of irradiation and chemotherapy. That this is in fact a feasible option is demonstrated by the third patient presented. It demonstrates the power of applying the current markers for diagnosis of the premalignant lesions of type II GCTs. In fact, reevaluation of the biopsy of this patient, taken at three years of age, showed coexpression of OCT3/4 and TSPY in germ cells located on the basal lamina. These cells are referred to as pre-CIS, from which CIS will develop. Proper identification of the risk factors for type II GCTs, in particular related to DSD and TDS, will increase the possibility to identify patients at risk for malignancy at an early age, allowing application of limited-harmful treatment protocols.

OCT3/4 expression is most likely related to the survival of the germ cells [24], while the role of TSPY is less clear. It has been suggested to be related to cell-cycle regulation [25–27]. In addition, SCF is informative to diagnose CIS and GB, especially to distinguish CIS from germ cells delayed in their maturation [28]. Of interest in this context is the linkage of specific single nucleotide polymorphisms with development of type II GCTs in the general Caucasian population, including involvement of SCF [15, 16]. However, the impact of these risk alleles in the DSD populations remains to be investigated.

The left testis of patient 1 and the right testis of patient 3 are still *in situ* at a scrotal localization. For the first patient available hormonal data indicated suboptimal testicular function (high FSH, low inhibin, testosterone low normal range). In spite of treatment by prophylactic irradiation, and absence of metastasis (based on routine examinations), the patient is under close surveillance because of a minor elevated AFP level. No hormonal indications for testicular dysfunction could be observed in patient 3, while no data were available for patient 2.

Recently two families have been independently reported showing an overlap between DSD and TDS. One family showed two sisters with XY sex reversal, gonadal dysgenesis, and GB, and the other family included one daughter with a mosaic karyotype and GB. All patients showed a *SRY* mutation, inherited from the father, being mosaic. The fathers of both families presented with TDS, one with oligoasthenozoospermia, and a testicular SE, the other with hypospadias, cryptorchidism, oligoasthenozoospermia, and a testicular SE as well [29, 30]. In other words, TDS and DSD form a continuum, which is informative to identify individuals at risk for type II GCTs.

As indicated, the two patients demonstrated here with a type II GCT, and proven metastasized cancer in one, at time of diagnosis, also show the value of identification of parameters known to be related to TDS and DSD, including cryptorchidism, hypospadias, and the presence of GB (in the latter). Based on these characteristics, these two patients would have been diagnosed as 46,XY DSD, disorder of testicular development, 1: partial gonadal dysgenesis [1], being at increased risk for development of a malignant type II GCT. Feasibility of early diagnosis, leading to the prevention of development of an invasive, and possibly even a metastatic cancer, is clearly demonstrated by the third patient.
